# Irinophore C™, a lipid nanoparticle formulation of irinotecan, abrogates the gastrointestinal effects of irinotecan in a rat model of clinical toxicities

**DOI:** 10.1007/s10637-014-0138-x

**Published:** 2014-07-27

**Authors:** Dawn N. Waterhouse, Brent W. Sutherland, Nancy Dos Santos, Dana Masin, Maryam Osooly, Dita Strutt, Christina Ostlund, Malathi Anantha, Natashia Harasym, Irina Manisali, Mohamed Wehbe, Marcel B. Bally, Murray S. Webb

**Affiliations:** 1 Experimental Therapeutics, BC Cancer Agency, Vancouver, BC Canada; 2 Department of Pathology and Laboratory Medicine, University of British Columbia, Vancouver, BC Canada; 3 The Centre for Drug Research and Development, 2405 Wesbrook Mall, 4th floor, Vancouver, BC V6T 1Z3 Canada; 4 The Faculty of Pharmaceutical Sciences, University of British Columbia, Vancouver, BC Canada

**Keywords:** Irinotecan, Nanoparticle, Gastrointestinal toxicity, Pharmacokinetics, 5-FU, Diarrhea

## Abstract

Irinotecan is a water-soluble camptothecin derivative with clinical activity against colorectal and small cell lung cancers and is currently a standard of care therapeutic in the treatment of colorectal cancer in combination with 5-fluorouracil. One of the major clinical issues limiting the use of irinotecan is gastrointestinal toxicity manifested as life-threatening diarrhea which is reported in up to 45 % of treated patients. The studies summarized here tested, in a rat model of irinotecan-associated gastro-intestinal toxicity, whether a lipid nanoparticle formulation of irinotecan, Irinophore C™, mitigated early-onset or late-onset diarrhea when given at doses equivalent to unformulated irinotecan that engenders both early- and late-onset diarrhea. Specifically, rats administered intravenously on two consecutive days with unformulated irinotecan at 170 mg/kg then 160 mg/kg experienced transient early-onset diarrhea after each administration and then experienced significant late-onset diarrhea peaking 4 days after treatment. Irinophore C™ given at the identical dose and schedule did not elicit either early- or late-onset diarrhea in any animals. When Irinophore C™ was combined with 5-fluorouracil there was also no early- or late-onset diarrhea observed. Histopathological analysis of the gastro-intestinal tract confirmed that the effects associated with irinotecan treatment were absent in rats given Irinophore C™ at the identical dose. Pharmacokinetic analysis demonstrated significantly higher systemic concentrations of irinotecan in rats given the nanoparticle formulation compared to those given unformulated irinotecan. These results demonstrate that the Irinophore C™ formulation is significantly less toxic than irinotecan, used either as a single agent or in combination with 5-fluorouracil, in a rat model of irinotecan-induced gastrointestinal toxicity.

## Introduction

Irinotecan is a water-soluble camptothecin derivative with clinical activity against colorectal and small cell lung cancers and is a standard of care therapeutic in the first-line treatment of colorectal cancer in combination with 5-fluorouracil (5-FU). Irinotecan also has demonstrated activity in lung [1], gastric [2], pancreatic [3] and ovarian [4] cancers and lymphoma [5]. Irinotecan has a complex pharmacological profile, and is predominantly converted by carboxylesterases to a more active form, 7-ethyl-10-hydroxycamptothecin (SN-38). Both irinotecan and SN-38 cause irreversible DNA damage via stabilization of the cleavable complex that forms between topoisomerase I and DNA during replication. While the activities of irinotecan and SN-38 activities are dependent on maintenance of the lactone ring conformation, physiological pH favors the formation of the less active carboxylate structure. This property makes irinotecan a strong candidate for drug carrier technology, such as the lipid nanoparticle formulation Irinophore C™, in which encapsulation stabilizes the lactone ring structure and extends the circulation lifetime of both irinotecan and SN-38 [6–8]. The pharmacokinetics and biodistribution of this formulation in tumor-free and tumor-bearing mice has been reported previously [7–10]. Irinophore C™ achieves significantly enhanced anticancer efficacy, compared to free irinotecan, in a number of human xenograft tumor models [7, 9, 10].

Increasing the therapeutic activity of irinotecan, as can be achieved using nanoparticulate drug carriers, will only be clinically important if the toxicities of the drug are comparable or reduced. Patients treated with irinotecan-based chemotherapy are at high risk for developing gastrointestinal (GI) mucositis, manifested as severe diarrhea, which occurs in 45 to 80 % of cases [11–13]. This side effect often leads to delay in treatment, dose reductions, or treatment cessation; all of which can compromise treatment outcomes. Two specific forms of GI toxicity are associated with irinotecan therapy. The first is early onset diarrhea associated with cholinergic symptoms such as cramps, diarrhea, salivation, visual disturbances and lacrimation [11]. A more insidious toxicity is late onset diarrhea, which manifests at the third day post-treatment and may be life threatening [12]. One assessment of irinotecan induced late onset diarrhea [11] showed that 82 % of patients experienced this toxicity while others report incidence of 50–80 % [12]. Acute toxicity assessments in mice and rats have suggested that Irinophore C™ may be 2- to 3-fold less toxic than free irinotecan based on clinical observations, behavioral changes and weight loss (unpublished observations). These preliminary studies, however, did not address the specific problem of late onset gastrointestinal toxicity that is most relevant in the clinical setting. Furthermore, since results from our lab that have shown higher plasma concentrations of SN-38 over extended times when irinotecan is administered as Irinophore C™ [8], it might be expected that Irinophore C™ treatment could increase the severity of late onset gastrointestinal toxicity compared to free irinotecan.

In the studies reported here, we compared the ability of Irinophore C™ and irinotecan to illicit late onset diarrhea in a rat model designed to mimic the clinical presentation of this GI toxicity (adapted from Trifan et al. [14]). Further, we have also compared Irinophore C™ and irinotecan when co-administered with 5-FU as is mostly commonly used for the treatment of colorectal cancer. The results show that significant adverse GI toxicities, specifically both early and late onset diarrhea, associated with irinotecan or irinotecan plus 5-FU treatment are not observed when irinotecan is given in the nanoparticle formulation Irinophore C™.

## Methods

### Lipid nanoparticle preparation

1,2-distearoyl-*sn*-glycero-3-phosphocholine (DSPC, Lipoid, Newark, NJ) and cholesterol (Avanti Polar Lipids, Alabaster, AL), in a DSPC/cholesterol ratio of 55/45 (mol/mol), were used as follows: lipids were dissolved at the DSPC/cholesterol 55/45 M ratio in 2,030 mL of anhydrous ethanol with heating to 40 °C, sterile filtered and then mixed with 21,000 mL of a degassed 300 mM copper sulphate solution. During this initial dispersion step, the ethanol lipid solution was maintained at ~50 °C and the copper sulphate was maintained at ~70 °C. This lipid suspension was concentrated to a final volume of 800 mL using an ultrafiltration hollow fibre cartridge system (GE Healthcare) then extruded through stacked 0.2 and 0.1 μm filters in an 800 mL thermobarrel extruder (Extruder™, Northern lipids, Vancouver, BC, Canada). The resultant nanoparticles had a mean diameter of 90–115 nm as determined by Phase Analysis Light Scattering methods (ZetaPALS, Brookhaven Instruments Corp., Holtsville, NY). To remove ethanol, the external buffer was exchanged with a 300 mM copper sulphate solution using an ultrafiltration hollow fibre column (GE Healthcare). Subsequently, the external buffer was exchanged to a sucrose/HEPES/EDTA buffer pH 7.4 (300 mM sucrose, 20 mM HEPES, 15 mM EDTA) and diluted to a final volume of 3578.0 mL to allow for irinotecan drug loading.

### Loading of irinotecan into preformed DSPC/cholesterol nanoparticles

Prior to drug loading, the cholesterol concentration was quantified by an HPLC method and the measured cholesterol concentration (13.26 mg/mL) was used to determine the required amounts of A23187 and irinotecan to be added to the nanoparticles. A23187 is a divalent metal ionophore that is used to facilitate the loading of irinotecan into the Irinophore C™ nanoparticle using the procedure described below and in [6]. HPLC analysis of cholesterol was performed using a C18 Symmetry shield column (4.6 × 75 mm, 3.5 μm particle size, Waters Mississauga ON) and an isocratic elution of 92 % methanol and 8 % of water. Sample preparation was done by extraction using CHCl_3_:methanol (1:1, vol:vol) solvent, evaporating the organic layer and reconstituting the residue with 100 % methanol. (+)−4-cholesTen-3-one (3ONE) was used as internal standard. Samples (20 μL) were injected onto the column and eluted at a flow rate of 1.5 mL/min. Sample temperature was maintained at 15 °C, column temperature at 30 °C. Cholesterol was measured based on the integration of the peak area using a Waters 996 photodiode array (PDA) detector (Waters, Mississauga, ON) set with wavelength of 202.8 nm. The retention time for cholesterol was 17 min and (+)−4-cholesTen-3-one (3ONE) was 13 min. Measurements were done in triplicate.

Based on the cholesterol concentration determined as described above, 14.52 mL of A23187 (calcimycin; Sigma, Oakville, ON, Canada; 2 mg/mL (3.8 mM) solution in 100 % ethanol) was pre-incubated with the nanoparticle suspension (at 1.748 μg/mg cholesterol) at 50 °C for 30 min. Subsequently, 575.4 mL of irinotecan hydrochloride trihydrate (Sichuan Guanghan Biotech) solution composed of irinotecan (25.6 mg/mL), sorbitol (45 mg/mL) and lactic acid (0.09 mg/mL) in water, was added to the nanoparticle suspension (at a drug-to-lipid ratio of 0.2:1 (mol:mol) and drug loading into the nanoparticles was allowed to proceed at 50 °C for 60 min.

After irinotecan loading, the solution was cooled to 40 °C, concentrated by tangential flow to 950 mL, buffer exchanged with glycerine/TRIS (10 % w/w glycerine/0.435 % w/w (~40 mM) TRIS) using an ultrafiltration hollow fibre column (GE Healthcare) and quantified for irinotecan drug concentration. Because fluorescence measurements are more sensitive than UV measurements for irinotecan, UV detection of irinotecan for quantification purposes was not preferred. Therefore, irinotecan concentration in the product concentrate was measured using HPLC set-up with a RP C18 Symmetry shield column (size 250 × 4.6 mm, particle size 3.5 μm) and C18 Symmetry shield guard column (Waters, Mississauga, ON). An isocratic elution of 78 % of mobile phase A and 22 % of mobile phase B was used with mobile phase A composed of 75 mM ammonium acetate and 7.5 mM tetrabutylammonium bromide adjusted to pH = 6.4 with glacial acetic acid and mobile phase B of 100 % acetonitrile. An irinotecan standard curve was prepared by serial dilution in 4 % acidified methanol. Sample dilutions were also made in 4 % acidified methanol. Samples (10 μL) were injected onto the column and eluted at a flow rate of 1 mL/min. Prior to injection all standards and samples were maintained at 4 °C. Column temperature was set at 35 °C. Irinotecan was quantified based on the standard using a Waters 2475 multi-wavelength fluorescence detector (Waters, Mississauga, ON) set at 370 nm excitation and 425 nm emission. The retention time for irinotecan was 12 min. Measurements were done in triplicate. The product concentrate was then diluted to 407 mL with the glycerine/TRIS buffer to achieve a final irinotecan concentration of 11 mg/mL. The resultant Irinophore C™ product was aseptically sterile filtered and filled into 10 mL vials (5 mL per vial). Vials were frozen at−80 °C until required. It should be noted that this large scale batch of approx 400 mL of final sterile Irinophore C™ product was generated to support a wide variety of studies including those described here as well as other product stability and GLP related studies.

### Animals

Female Sprague–Dawley rats were purchased from Harlan Laboratories, Inc. (Indianapolis, IN). Rats were housed in pairs and maintained in sterilized ventilated filter-topped cages in a specific pathogen-free barrier facility (Animal Resource Center (ARC) at the British Columbia Cancer Agency’s Vancouver Cancer Research Centre); irradiated food and chlorinated treated water were given ad libitum. Animals were acclimatized to their holding room for a minimum of 5 days prior to initiating the studies. Sentinel animals from holding rooms were tested for common pathogens, and results were negative for the duration of this experiment and for a minimum of 18 months prior. All studies were completed under an animal care protocol approved by the Institutional Animal Care Committee (IACC). The IACC for studies conducted at ARC is operated by the University of British Columbia and operates in accordance to the Canadian Council of Animal Care (CCAC). The ARC is a CCAC audited facility.

### Establishment of late onset diarrhea model

The model of irinotecan-induced diarrhea used here was modified from that described by Trifan et al. [14]. To establish this model in our facility, groups of rats (*n* = 2) were treated i.v. (by the tail vein) on two consecutive days with 160, 170 or 180 mg irinotecan/kg or at 170 mg/kg on day 1 and then at 160 mg/kg on day 2. Irinotecan was infused using a syringe pump at a maximal infusion rate of 2.5 mL/min/kg; a total volume of approximately 8 mL was administered over a 15 min time period. A group of two rats was given 5 % dextrose in water (D5W) by infusion to establish that the infusion rate and volume could be safely given. Blinded observations were conducted daily until day 10 to confirm recovery from the adverse effects caused by treatment. Diarrhea was scored twice per day (approx. 7 am and 4 pm) using the following scale: 0 (normal stool or absent), 1 (slight wet/soft stool), 2 (more than slight but less than moderate diarrhea), 3 (moderate diarrhea: wet and unformed stool with moderate perianal staining of the coat), 4 (moderate diarrhea; severe anal staining) and 5 (severe diarrhea: watery stool with severe perianal staining of the coat). Assessments were conducted on fresh visible stool samples and cages were changed daily. Body weights and clinical signs were also observed and recorded daily. Clinical signs assessed in addition to diarrhea included: fur/skin-related signs, behavioural or motor function-related signs, whole body signs, limb or paralysis-related signs, breathing-related signs and bodily function-related signs. These clinical signs were assigned scores of 0 to 5. Any rat having an individual observation with severity score of 5, or a combined observation score of 10, was euthanized by CO_2_ asphyxiation (except where noted). All scoring was conducted under a Standard Operating Procedure (SOP) and the observer trained under this SOP was blinded to the study. A single observer was used to record data through the course of the experiment.

### Histopathology assessment

GI tissue including the duodenum, jejunum, ileum and the colon were collected 3 days after the last dosing day. Approximately 2 in. of each section of the intestinal tract were excised and any contents were gently removed by flushing with phosphate-buffered saline (PBS). Each section was then cut transversely into 2 halves and then one of the halves was cut longitudinally. Both tissue pieces were rinsed in PBS and then fixed in Molecular Fixative (Somagen, Edmonton, AB). Fixed tissues were sent to Wax-it Histology Services Inc. (Vancouver, BC, Canada) and embedded in paraffin. Cross sections of the intestinal tract (5 μm) were cut and mounted on slides and stained with hematoxylin and eosin (H&E). Slides were then sent to Animal Pathology Services Ltd. (Alberta, Canada) and were examined by a board certified veterinary pathologist. The pathologist was blinded to the experimental group assignments of each animal. Slides were scanned with the Panoramic MIDI (3DHISTECH, Budapest, Hungary) and images created using Panoramic Viewer from 3DHISTECH, version 1.5.

### Comparison of irinotecan with Irinophore C™ in a late onset diarrhea model

Groups of 12 female Sprague–Dawley rats were administered (by intravenous infusion as described above) with D5W, irinotecan or Irinophore C™ at 170 mg/kg (day 1) and 160 mg/kg (day 2). Diarrhea and clinical observations were scored daily for 10 days post administration. Four animals from each treatment group were sacrificed 3 days post administration for histological assessment of intestine and colon.

### Comparison of irinotecan plus 5-FU versus Irinophore C™ plus 5-FU in a late onset diarrhea model

Groups of 8 female Sprague–Dawley rats were treated with a combination of irinotecan or Irinophore C™ at 150 mg/kg by intravenous infusion on day 1 and day 2 and with 5-FU at 15 mg/kg by slow bolus intravenous injections on day 1 and day 2. The administration of 5-FU was performed immediately before the administration of irinotecan or Irinophore C™. In these studies, rats were observed daily for 20 days for clinical assessment and scoring of diarrhea as described above. Control groups consisted of each test article or saline administered (as described above) as a single agent. Body weights, in-life observation scores and diarrhea assessments were individually subjected to a 2-way mixed model ANOVA with treatment condition as a between subject factor and time (days) as the within-subject factor. Between group differences were analyzed using Bonferroni post hoc analysis.

### Pharmacokinetic study and bioanalysis

For the pharmacokinetic studies, the rats were administered as described above using doses of 170 mg/kg on day 1 and 160 mg/kg on day 2. Blood was collected by saphenous vein or by cardiac puncture at the final time point. Blood was collected into EDTA-containing Microtainer® tubes and the plasma was recovered after centrifugation.

For sample extraction, 150 μL of 4 % acetic acid in methanol was added to aliquots of 50 μL plasma. This was centrifuged for 15 min at 4 °C and 13,500 rpm (17,110 rcf). Following centrifugation, 100 μL supernatant was transferred to tubes containing 1,400 μL acetic acid in water. This was then mixed on a vortex mixer and transferred to solid phase extraction wells using moderate vacuum. Analytes were eluted with 400 μL 4 % acetic acid in methanol then evaporated to dryness. Sample residues were reconstituted into 150 μL of 100 ng/mL camptothecin (Sigma; internal standard) in 4 % acetic acid in acetonitrile. 2 μL of reconstituted samples were injected onto UPLC-MS/MS for analysis. Standard curves were generated using plasma samples spiked with irinotecan HCl (Sigma).

Analysis was performed on a Waters Acquity UPLC system using a Waters Acquity BEH C18 1.7 μm, 2.1 mm × 50 mm column maintained at 30 °C and Waters TQD and PDA detectors. Mobile phase A was water with 0.1 % formic acid. Mobile phase B was acetonitrile with 0.1 % formic acid. A gradient of 5 to 70 % B in 2.5 min, wash at 98 % B for 0.5 min, back to initial conditions in 1.3 min was used. Pharmacokinetic parameters were estimated using a non-compartmental analysis with Phoenix WinNonlin (v. 6.2).

## Results

### Establishment of late onset diarrhea model

**Fig. 1:**
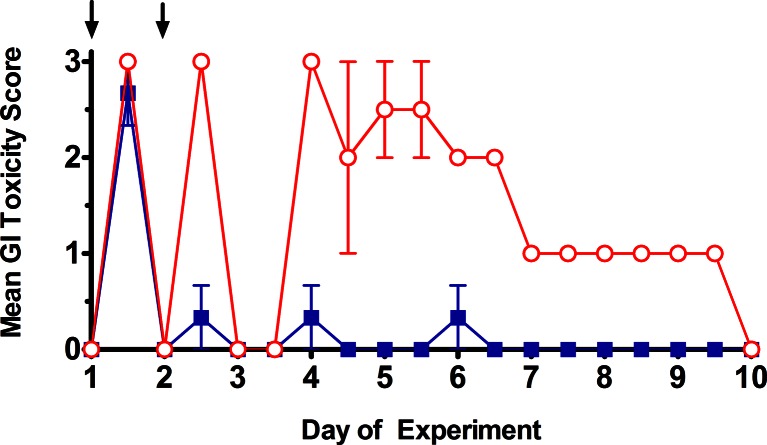
GI toxicity (diarrhea) scores for rats administered with irinotecan at either 160 mg/kg on days 1 and 2  or at 170 mg/kg on days 1 and 2 . Administrations are indicated by arrows. Symbols represent the means ± standard error of 3 rats in the irinotecan 160 mg/kg group and 2 rats in the irinotecan 170 mg/kg group. All data was collected in blinded fashion

The model of camptothecin-induced late onset diarrhea used here was adapted from that described previously [14]. To assess GI toxicities associated with D5W (control) or irinotecan administration, female Sprague–Dawley rats had diarrhea scored twice daily as described in the Methods. Scores were tabulated and averaged for the different treatment dosages of 160 mg/kg (Fig. 1), 170 mg/kg (Fig. 1) or 180 mg/kg on day 1 and day 2 (not shown). The D5W control treatment rats did not exhibit any signs of diarrhea, having an average group score of 0 throughout the study (data not shown). Rats dosed with irinotecan at 160 mg/kg on two consecutive days exhibited partial early-onset diarrhea, but no late-onset diarrhea. This is shown by the high GI toxicity score on day 1.5 (the afternoon measurement of day 1 treatment) with a mean score of 3, a very low GI toxicity score of <0.5 after the second dose (day 2.5; afternoon measurement of day 2) and negligible GI toxicity (scores >1.0) observed over the remaining time course. Rats given irinotecan at the higher dose of 170 mg/kg exhibited early onset diarrhea on both administrations days (days 1.5 and 2.5, Fig. 1). In the absence of any further treatment, animals given 170 mg/kg irinotecan exhibited signs of late stage diarrhea starting on day 4 and persisting until day 7. These signs included GI toxicity scores of between 1 and 3 through day 9 (Fig. 1) and clinical observation scores ranging from 5 to 7.5 through day 7 (not shown). This second toxicity phase was associated with more severe effects where mean body weight loss of almost 10 % was noted on days 5 and 6 of the experiment (data not shown). By the end of this experiment the animals had almost completely recovered with the exception for some mild porphyrin staining on the neck area.

These preliminary studies demonstrated that two consecutive doses of 160 mg/kg irinotecan were not sufficient to cause late onset diarrhea and while two consecutive doses of irinotecan at 170 mg/kg did induce late onset diarrhea, however these animals almost reached humane endpoints specified in the protocol. As indicated, these preliminary studies also assessed toxicity associated with two consecutive dose of 180 mg/kg irinotecan. This dose resulted in toxicities severe enough to warrant euthanasia (data not shown).

### Irinotecan compared with Irinophore C™ in a late onset diarrhea model

**Fig. 2:**
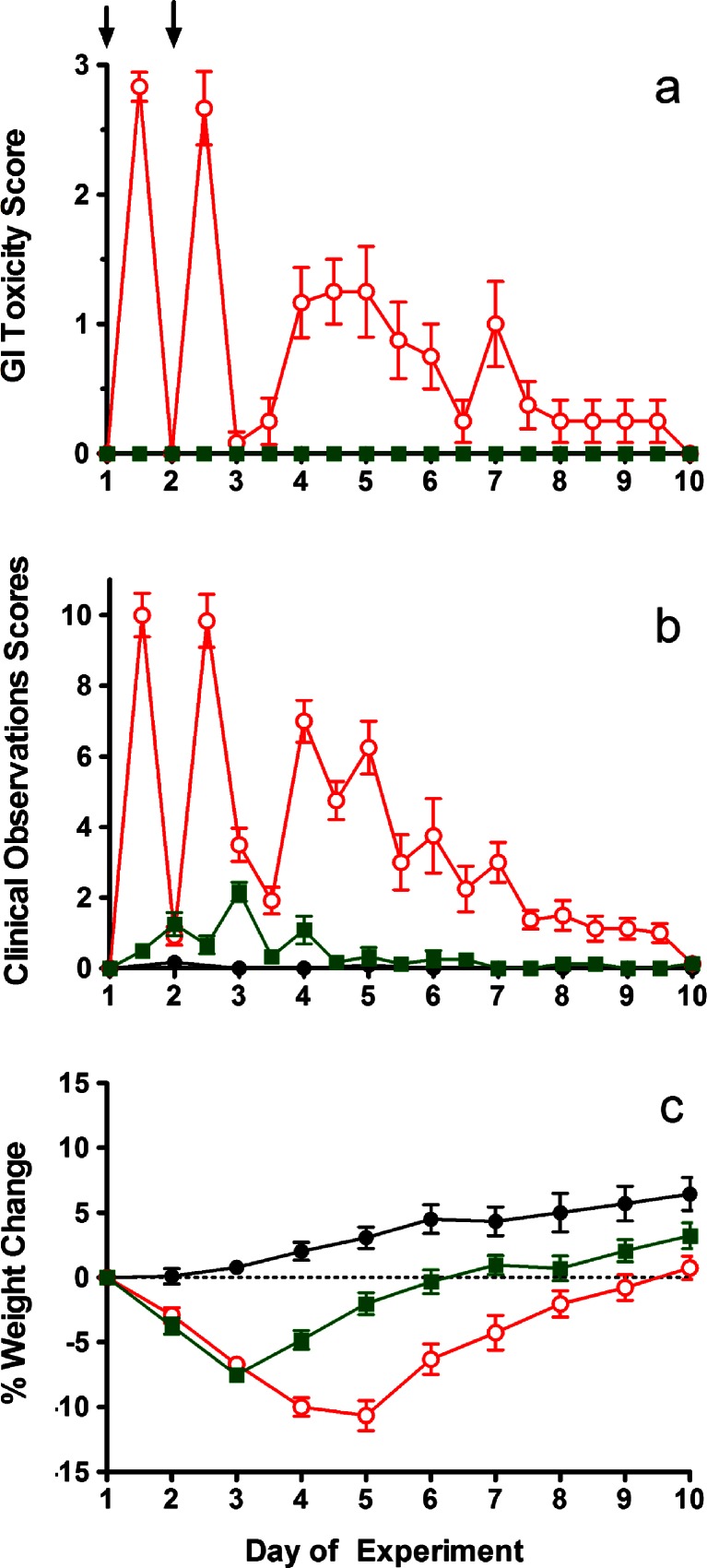
GI toxicity (diarrhea) score (**a**), total clinical observation scores (**b**) and body weight change as a percentage of weight on day 1 (**c**) for rats given D5W (equal volume to irinotecan) (●), irinotecan  or Irinophore C™  at 170 mg/kg on day 1 and 160 mg day 2. Administrations are indicated by arrows. Symbols represent the means ± standard error of 4 rats. All data was collected in blinded fashion

Based on the studies summarized above, subsequent studies used a treatment schedule consisting of 170 mg/kg administered on day 1 and 160 mg/kg on day 2. Rats were given either irinotecan or Irinophore C™ with inclusion of additional animals for histopathological assessments of the GI tract at the peak of late onset GI toxicity. Figure 2 summarizes the results for GI toxicity (Fig. 2a), clinical observations (Fig. 2b) and body weights (Fig. 2c).

Consistent with the model development work described above, administration of irinotecan resulted in early onset of GI toxicity with moderate diarrhea consisting of wet and unformed stool with moderate perianal staining of the coat. Average GI toxicity scores of 2.8 were recorded following the first injection and 2.7 following the second injection; these were observed in the afternoons of each infusion day (Fig. 2a). Late onset diarrhea was also observed in these animals, reaching a peak with an average GI toxicity score of 1.2 on day 5, approximately 72 h after the final dose of irinotecan. Rats given irinotecan also exhibited several clinical observations including abnormal gait, shaking and twitching, hunched posture while sitting and marked porphyrin staining on the neck and nose area. In this group mean clinical scores of 10 were observed in animals during the infusion days of the experiment and in the early onset of diarrhea phase. In some animals scores transiently in excess of 10 were noted, but after consultation with the clinical veterinarian and based on the preliminary study indicating that acute toxicities resolve rapidly, these animals were maintained on study. Scores subsequently decreased to approximately 2, but then peaked again during the late stage diarrhea phase (days 4–7) with mean clinical scores of 6–7 (Fig. 2b). By the end of the study (day 10) these animals had completely recovered. GI toxicities noted in the irinotecan treated animals were associated with mean body weight losses of just over 10 % on day 5 of the study (Fig. 2c).

When irinotecan was administered as the lipid nanoparticle formulation Irinophore C™ (Fig. 2), no GI toxicity was observed at any time during the study. Both early and late onset diarrhea were absent (Fig. 2a; *p* < 0.001, as compared with irinotecan treated animals). Rats given Irinophore C™ scored identical to, and were not statistically different from, the D5W control in terms of GI toxicity (Fig. 2a). However, administration of Irinophore C™ did result in small, but measurable, changes in clinical observations (Fig. 2b) and a 7 % loss in mean body weight (day 3) which was almost completely resolved by day 10 (Fig. 2c). Animals in the D5W control group did not show any signs of stress related to the infusion or handling. In-life clinical observation scores were 0 in these animals.

Necropsies were performed on all animals, with one group of animals euthanized on day 5 while the remaining animals were euthanized at the end of the study (day 10). No unusual findings were noted in animals given infusions with D5W. On day 5, the irinotecan treated rats had major GI abnormalities including distension, inflammation and swelling of the intestines and stomach. In contrast, those rats given Irinophore C™ only exhibited slightly inflamed intestines and had no other findings. All animals appeared normal on gross necropsy at the end of the study, 8 days after the last dose was given.

**Fig. 3:**
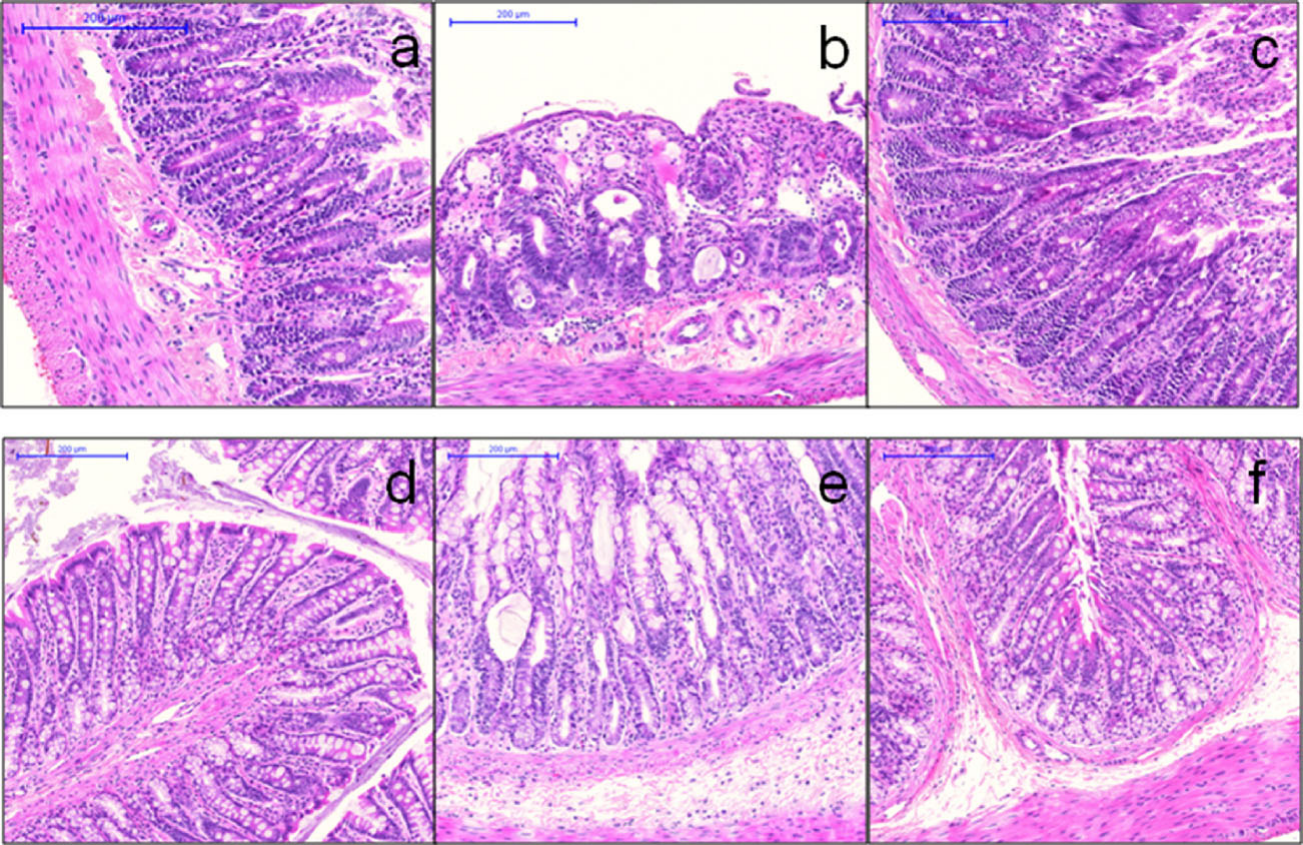
Hematoxylin and eosin stained sections of colon (a–c) and small intestine (duodenum; d–f) from rats given D5W (a, d), irinotecan (b, e) or Irinophore C™ (c, f). All images obtained with 10X objective. Note the shortening and fusion of villi, loss of villar epithelium with flattening of remaining epithelia cells, dilation of villar lymphatics, edema of mucosal lamina propria and congestion of capillaries in irinotecan-treated rat sections (b, e). Bars indicate 200 μm

GI tissue including parts of the duodenum, jejunum, ileum and the colon were collected from rats on day 5. Approximately 2 in. of each section of the intestinal tract were excised and any contents gently removed by washing and prepared for histological assessments as described in the Methods. H&E stained slides were provided to a board certified veterinarian pathologist who was blinded to the groups. Specific observations by the pathologist were recorded and these have been summarized in Table 1. For the irinotecan group, severity evaluations are provided in Table 2 and representative images of colon and duodenum are shown in Fig. 3.

**Table 1:** Histopathological findings in rats treated with D5W, irinotecan or Irinophore C™

Animal #	Organ	Observations
D5W control		
# 25, 26 & 27	Duodenum	Microscopically normal
	Jejunum	Microscopically normal
	Ileum	Microscopically normal
	Colon—ascending	Microscopically normal
	Colon—descending	Microscopically normal
	Colon—transverse	Microscopically normal
#28	Duodenum	Microscopically normal
	Jejunum	Microscopically normal
	Ileum	Cross section: Loss of the epithelial lining of the villi with an increase in the number of mononuclear inflammatory cells in the villi and focal areas of congestion of the capillaries at the tips of the villi. Longitudinal section: local hemorrhage in the serosa and congestion of villar capillaries is more general.
	Colon—ascending	Microscopically normal
	Colon—descending	Microscopically normal
	Colon—transverse	Microscopically normal
Irinotecan		
# 29	Duodenum	Microscopically normal
	Jejunum	Dilation of the lymphatics of the mucosa, particularly in the villi. Edematous lamina propria without increase in inflammatory cells in mucosa.
	Ileum	Microscopically normal
	Colon—ascending	Microscopically normal
	Colon—descending	Microscopically normal
	Colon—transverse	Microscopically normal.
# 30	Duodenum*	Alteration of the mucosal architecture: shortening and fusion of villi, loss of villar epithelium. Flattening of the remaining epithelial cells, dilation of the villar lymphatics, and edema of the mucosal lamina propria. Congested capillaries.
	Jejunum	Shortened and fused villi. Mild edema of villar cores.
	Ileum	Microscopically normal
	Colon—ascending	Mild autolysis
	Colon—descending	Microscopically normal
	Colon—transverse	Cross section: A number of crypts throughout the mucosa are widely dilated, with general secretion of fluid into the lumen. Flattened crypt epithelium. Mild edema of the lamina propria. Longitudinal section: epithelial damage with vascular reaction at several levels of the GI tract without associated inflammation.
# 31	Duodenum	There is congestion of some of the capillaries of villi, and edema of the lamina propria of such villi, especially at the tips.
	Jejunum	Changes in the ileum similar to those in the duodenum
	Ileum	Cross section: Shortening and fusion of villi, loss of villar epithelium with flattening of the remaining epithelial cells, wide dilation of the villar lymphatics, and edema of the mucosal lamina propria. Multiple instances of several villi to be fused with a single overarching layer of epithelial cells. Longitudinal section: flattened mucosal surface with no villi protruding into the lumen.
	Colon—ascending	Microscopically normal
	Colon—descending	Microscopically normal
	Colon—transverse	Microscopically normal
# 32	Duodenum	Complete disorganization of the normal architecture. Loss of villi and collapse of villar mucosa. The resulting mucosal lining consists of a series of widely dilated lymphatics from the villar cores mixed with a smaller number of dilated crypts.
	Jejunum	Microscopically normal
	Ileum	Microscopically normal
	Colon—ascending	Microscopically normal
	Colon—descending	Microscopically normal
	Colon—transverse	Microscopically normal
Irinophore C™		
# 33, 34 & 36	Duodenum	Microscopically normal
	Jejunum	Microscopically normal
	Ileum	Microscopically normal
	Colon—ascending	Microscopically normal
	Colon—descending	Microscopically normal
	Colon—transverse	Microscopically normal
# 35	Duodenum	There are two widely dilated crypts. One of these contains a large protein cast within which there are colonies of short rod shaped bacteria.
	Jejunum	Microscopically normal
	Ileum	Microscopically normal (large numbers of filamentous bacteria in lumen)
	Colon—ascending	Microscopically normal (large numbers of filamentous bacteria in lumen)
	Colon—descending	Microscopically normal
	Colon—transverse	Microscopically normal

**Table 2:** Histological lesion and severity summary for the irinotecan-treated rats. There were no lesions observed in the animals treated with Irinophore C™

Animal #	Group	Duodenum	Jejunum	Ileum	Colon
29	Irinotecan		+*		
30	Irinotecan	+++	++		++
31	Irinotecan	+	+	+++	
32	Irinotecan	+++			

* +, ++ and +++ indicate severity, from least to most severe

In the D5W control group, one animal had moderate changes in the ileum section of the intestine with loss of the epithelial lining of the villi, an increase in the number of mononuclear inflammatory cells in the villi and focal areas of congestion of the capillaries at the tips of the villi. In the longitudinal section there was additional local area of hemorrhage in the serosa and congestion of villar capillaries was more general. In these animals, the changes were considered to be “mild” to “very mild”. All other control animals were microscopically normal. Rats given irinotecan had changes in the intestine and the colon that, in general, consisted of vascular congestion and local edema through to alteration of the mucosal architecture with shortening and fusion of villi, loss of villar epithelium with flattening of the remaining epithelial cells and wide dilation of the villar lymphatics. Inflammation was not associated with these lesions. Qualitatively, such changes were similar in all affected animals, but quantitatively the severity varied considerably between individuals. Detailed histopathological observations for the irinotecan-treated animals are provided in Table 1 with a summary in Table 2. In rats given Irinophore C™, three of four rats were found to be microscopically normal on day 5. One rat in this group had dilated crypts in the duodenum, but was otherwise microscopically normal. This was discussed with the veterinarian pathologist after the study was unblinded and it was concluded that theses change did not match those seen in any of the rats given irinotecan; suggesting that the findings in this animal’s duodenum were incidental and unrelated to the study treatment.

### Comparison of Irinotecan plus 5-FU versus Irinophore C™ plus 5-FU in a late onset diarrhea model

Damage to the intestinal mucosa is a sequela of both 5-FU and irinotecan administration, leading to epithelia loss and subsequent increased fluid volume leaving the small bowel [15]. This in turn leads to diarrhea, the severity of which is amplified when the two agents are used in a combination therapy regimen. It is possible that the benefits in terms of reduced GI toxicity noted when irinotecan is given as the Irinophore C™ nanoparticle formulation could be lost when the formulation is combined with 5-FU. To test this, we also assessed the GI toxicity of 5-FU when used in combination with either irinotecan or Irinophore C™.

The 5-FU dose of 15 mg/kg on each treatment day was determined in a preliminary dose-range finding study in which irinotecan was given at 150 mg/kg on day 1 and day 2 and 5-FU given on each day at doses of 10, 15, 20 or 25 mg/kg. This study (data not shown) indicated that the 15 mg/kg dose of 5-FU, used in combination with irinotecan at 150 mg/kg on day 1 and day 2, elicited both early and late onset diarrhea and could be conducted within humane endpoints. The lower (10 mg/kg) dose of 5-FU did not elicit late onset diarrhea in combination with irinotecan and both higher doses of 5-FU (20 and 25 mg/kg) were not tolerated. Therefore, the combination of 150 mg/kg irinotecan plus 15 mg/kg 5-FU, both given on day 1 and day 2, was selected for the comparative study of irinotecan and Irinophore C™.

**Fig. 4:**
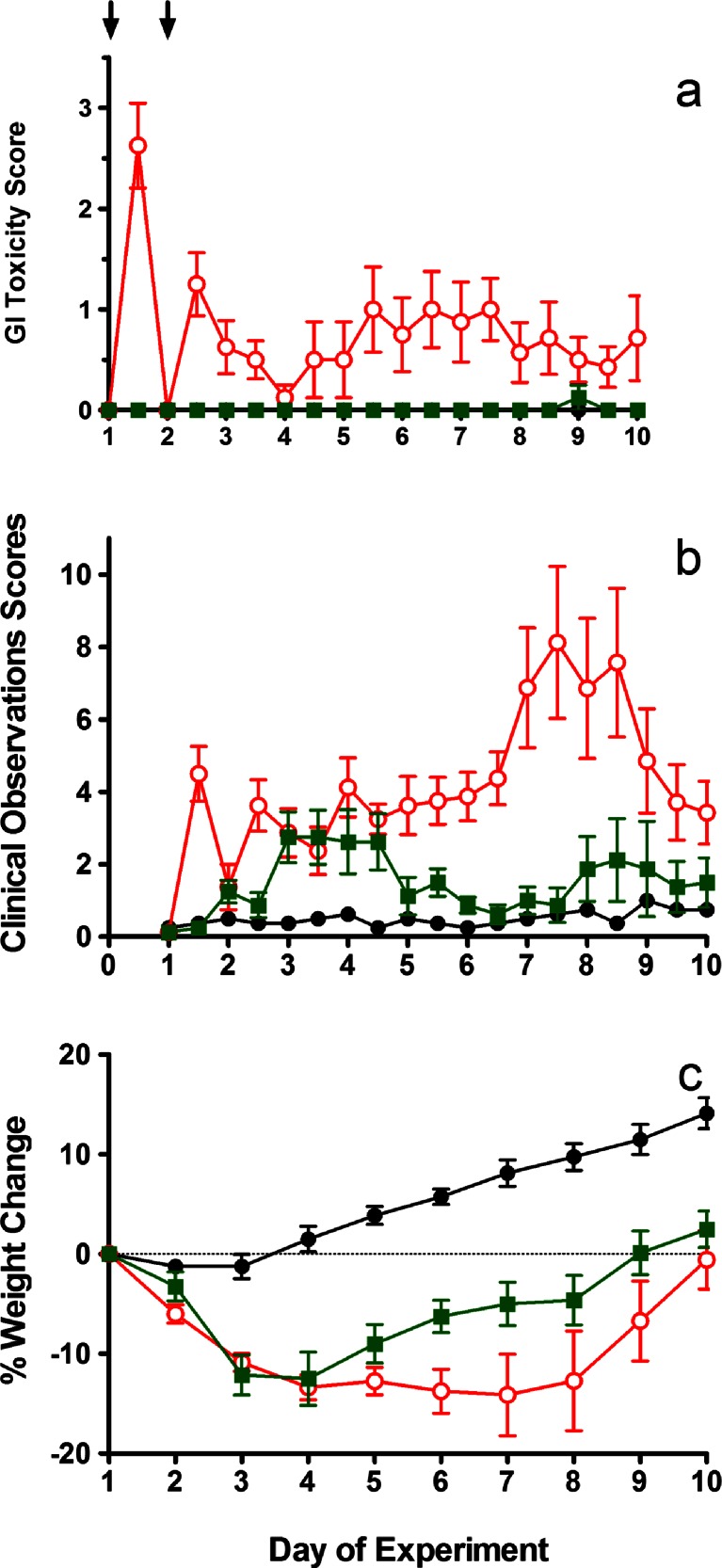
GI toxicity (diarrhea) score (**a**), total clinical observation scores (**b**) and body weight change as a percentage of weight on day 1 (**c**) for rats given saline (●), irinotecan at 150 mg/kg on days 1 and 2 plus 5-FU at 15 mg/kg days 1 and 2  or Irinophore C™ at 150 mg/kg days 1 and 2 plus 5-FU at 15 mg/kg on days 1 and 2 . Administrations are indicated by arrows. Symbols represent the means (±standard error) of 7–8 rats

Similar to the studies described above evaluating single agent irinotecan or Irinophore C™, these treatments were associated with reactions (twitching, shaking, poor gait and respiratory issues) that started 2–3 min after the onset of the infusion, continued for the duration of the 15 min infusion, but resolved within 1 h. GI toxicity and clinical observation scores, along with % change in body weight for the in-life portion of these studies, are summarized in Fig. 4. Rats given vehicle control (saline) on day 1 and day 2 did not experience any signs of GI toxicity (Fig. 4a) or changes in clinical observations (Fig. 4b) at any time during the study. Similarly, animals given 5-FU alone at 15 mg/kg showed no signs of GI toxicity or any changes in clinical observations (data not shown). The GI toxicity data indicated that a combination of irinotecan (150 mg/kg on day 1 and day 2) plus 5-FU (15 mg/kg on day 1 and day 2) was not well tolerated as reflected by a weight loss nadir of−7.7 %, (Table 3), significant clinical observation scores on days 7–9 and both early onset and late onset diarrhea (Fig. 4a). In marked contrast, treatment with Irinophore C™ (150 mg/kg on day 1 and day 2) in combination with 5-FU (15 mg/kg on day 1 and day 2) had no significant GI toxicity scores (Fig. 4a). These GI toxicity scores of the irinotecan plus 5-FU group and of the Irinophore C™ plus 5-FU group, given at the equivalent dose and schedule, were significantly different at both early onset timepoints (days 1 and 2; *p* < 0.001) and late onset timepoints (days 5.5–7.5; *p* < 0.05). There was, however, significant weight loss in the Irinophore C™ plus 5-FU group (nadir of−6.6 % on day 4; Table 3) and the clinical observation scores for this group were comparable to the irinotecan plus 5-FU group on days 3–4.

**Table 3:** Weight loss (average at nadir) and survival by study end for rats given saline, 5-FU, irinotecan, Irinophore C™, irinotecan plus 5-FU or Irinophore C™ plus 5-FU

Dose (mg/kg) on day 1 & day 2			Weight loss (%) at Nadir	Nadir loss (day)	Survival
Irinotecan	Irinophore C™	5-FU			
–	–	–	−0.7	3	100 % (8/8)
–	–	15	−1.8	2	100 % (8/8)
150	–	–	−7.5	5	100 % (8/8)
–	150	–	−5.7	3	100 % (8/8)
150	–	15	−7.7	7	87 % (7/8)
–	150	15	−6.6	4	100 % (8/8)

On necropsy, animals given irinotecan in combination with 5-FU had enlarged cervical and mesenteric lymph nodes. The latter were red in color. The spleen and liver exhibited a granular appearance, the intestines appeared slightly inflamed, thymus was small and partially reddish and the lungs appeared dark. Necropsy observations for these animals indicated a wide range of toxicities associated with the combination of 5-FU plus irinotecan that were not present in animals given either irinotecan or 5-FU as single agents. Necropsy observations of all animals given Irinophore C™ in combination with 5-FU at the end of the study suggested only minor changes: a marbled appearance on the kidney, swollen glands in the urogenital area of one animal and a malformed spleen in the same animal. Importantly, the exacerbated necropsy findings associated with the combination of 5-FU and irinotecan were not seen when 5-FU was combined with Irinophore C™. The ability of Irinophore C™ to abrogate the early and late onset toxicities elicited by irinotecan when the drug is given at equivalent doses in the free form, in the presence and absence of 5-FU, provides evidence suggesting that this dose-limiting toxicity may be reduced or eliminated in patients treated with this lipid nanoparticle formulation of irinotecan.

### Pharmacokinetics of Irinotecan and Irinophore C™

To complete these studies, an evaluation of the pharmacokinetics of irinotecan and Irinophore C™ was completed in rats. Irinotecan was administered at a dose and schedule that elicited both early and late onset diarrhea and Irinophore C^TM^ was given at the equivalent dose and schedule (intravenous infusions at a dose of 170 mg/kg on day 1 and at 160 mg/kg on day 2). Plasma concentrations of irinotecan were measured up to 96 h after the start of dosing and pharmacokinetic parameters estimated using a non-compartmental analysis.

When irinotecan was administered intravenously as the free drug, it was rapidly cleared from the circulation of rats with an estimated elimination half-life (T_½_) of 4.6 h, clearance (CL) of 873.0 mL/h/kg, a total AUC (AUC_0-∞_) of 378 μg∙h/mL and a volume of distribution (Vd) of 5820.1 mL/kg (Table 4). Area-under-the-curve and maximum plasma concentrations (C_max_) values for irinotecan were similar on day 1 (171.5 μg∙h/mL for AUC_0–24_) and day 2 (203.4 μg∙h/mL for AUC_24–48_) after the administration of free drug (Table 4), indicating negligible accumulation of drug on the second administration. This is consistent with the observation that the mean plasma concentrations of irinotecan 24 h after the day 1 administration of the free drug, 0.06 μg/mL, represented only 0.2 % of the concentration immediately after the day 1 administration (30 μg/mL) and only 0.3 % of the plasma concentration immediately after the day 2 infusion (22.1 μg/mL).

**Table 4:** Summary pharmacokinetic parameters for total irinotecan in rats after each administration of either irinotecan or Irinophore C™ at 170 mg/kg on day 1 then 160 mg/kg on day 2

Parameter	Irinotecan		Irinophore C	
	Day 1	Day 2	Day 1	Day 2
Dose (mg/kg)	170	160	170	160
C_max_ observed (μg/mL)	30.0	22.1	3,197	3,172
T_½_ (Elim.)* (h)	4.6		9.1	
AUC_0–24_ (μg∙h/mL)	171.5	–	33,083	–
AUC_24–48_ (μg∙h/mL)	–	203.4	–	42,548
AUC_0-∞_ (μg∙h/mL)	378		86,630	
Vd** (mL/kg)	5820.1		50.02	
CL** (mL/h/kg)	873.0		3.8	

* Estimated from the clearance curves after the second day of dosing, i.e., from 24 h to 72 h (irinotecan) or 96 h (Irinophore C™) after the study start

** Calculated based on AUC_0-∞_

**Fig. 5:**
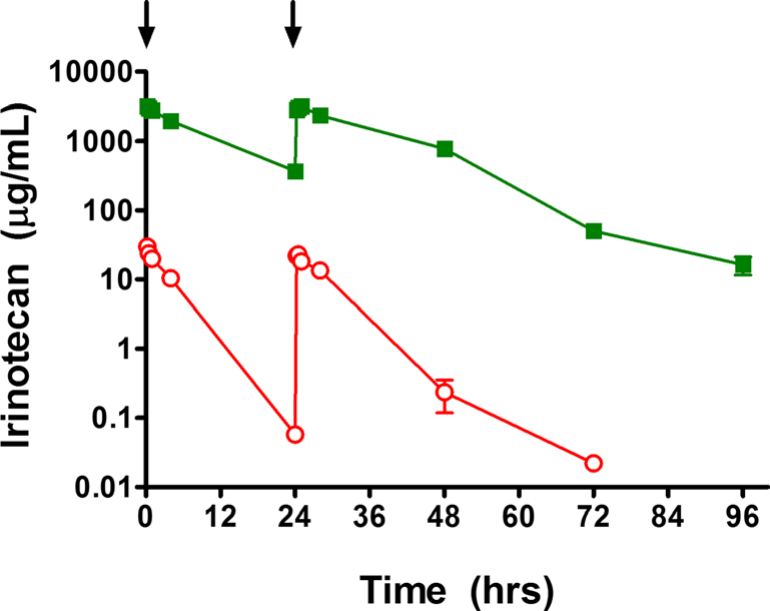
Plasma concentrations of irinotecan in rats at various times after the administration of either Irinophore C™  or irinotecan  at 170 mg/kg on day 1 (T = 0 h) then 160 mg/kg on day 2 (T = 24 h); arrows. Data represent the means (±standard error) of 6 animals

When irinotecan was administered intravenously in the Irinophore C™ nanoparticle formulation, the distribution and removal of the drug from the circulation was dramatically slower than that observed with free drug (Fig. 5). Specifically, the T_½_ was increased to 9.1 h, CL decreased by approximately 229-fold to 3.8 mL/h/kg, the total AUC corresponding increased by 229-fold up to 86,630 μg∙h/mL and the Vd decreased 116-fold to 50.02 mL/kg (Table 4). These changes were reflected by dramatic increases in the observed C_max_ of irinotecan in the plasma, 3,197 and 3,172 μg/mL on day 1 and 2, respectively. This represents 106- and 143-fold increases over the corresponding C_max_ values observed after the administration of free drug (Table 4). Although the C_max_ values after day 1 and day 2 administrations, 3,197 and 3,172 μg/mL respectively, were very similar, when the elimination curves were analyzed separately, the AUC values for irinotecan given as Irinophore C™ were significantly different on day 1 (33,083 μg∙h/mL for AUC_0–24_) and day 2 (42,548 μg∙h/mL for AUC_24–48_) (Table 4). This is consistent with the observation that the mean plasma concentrations of irinotecan 24 h after the day 1 administration of the Irinophore C™ nanoparticle, 369.6 μg/mL, represented 11.6 % of the concentration immediately after the day 1 administration (3,197 μg/mL) and 11.6 % of the C_max_ concentration immediately after the day 2 infusion (3,172 μg/mL). These observations indicate that the circulation lifetime of Irinophore C™ is sufficiently long that it is not entirely cleared 24 h after IV infusion and further daily administrations additively increase the systemic exposure to total irinotecan.

## Discussion

Late onset diarrhea is a major dose-limiting toxicity of irinotecan treatment in the clinic. A model of late onset diarrhea has been described previously [14]; a model in which diarrhea started approximately 48 h after the final dose of irinotecan and became more severe by day 5 before resolving by day 7–8. Although the doses and dose schedule of irinotecan needed to elicit early and late onset diarrhea in rats are different from those used therapeutically in humans, in both rat models and in humans early diarrhea occurs within 24 h of irinotecan administration and late diarrhea occurs more than 24 h after drug administration. We sought to reproduce this model in Sprague–Dawley rats and determine whether irinotecan-based GI toxicity was changed when the drug was administered as Irinophore C™. In the present study a reproducible model of late onset diarrhea is described however the doses needed to achieve late onset diarrhea were different than those reported by Trifan et al. [14]. In this study, irinotecan was dosed at 170 mg/kg on day 1 followed by 160 mg/kg on day 2. Importantly, very small increases in the dose of free irinotecan were sufficient to produce toxicities warranting euthanasia.

Using this model of GI toxicity, Irinophore C™, when given at equivalent doses to the maximum tolerated dose of free irinotecan, exhibited no GI toxicity. Evidence to support this included direct assessments of diarrhea (GI toxicity score) as well as clinical observations and weight of animals given irinotecan or Irinophore C™ as compared to animals given saline. For example, there were no measurable increases in GI toxicity seen in rats given Irinophore C™ whereas animals given irinotecan exhibited acute toxicity scores of almost 3 and late onset scores of greater than 1 (see Fig. 2). The absence of GI toxicity in rats given Irinophore C™ was corroborated by histological evaluation of the intestines; rats given free irinotecan exhibited significant histological changes while there were only minor observations in rats treated with Irinophore C™. It should be noted that Irinophore C™ did cause some toxicities in rats at the doses used here as shown by a nadir weight loss of almost 7 % on day 3 of the study (1 day after the last dose of Irinophore C™) and minor, but reproducible changes noted on necropsy. At the end of these studies (day 10) animals given irinotecan or Irinophore C™ appeared essentially normal. Importantly, the reduction of GI toxicity that occurred when irinotecan was given as the Irinophore C™ nanoparticle also occurred when given in combination with 5-FU. Data from our lab has shown in numerous cancer models that Irinophore C™ is much more efficacious then irinotecan, even when the Irinophore C™ is given at a dose equal to, or lower than, that of the free drug [7, 9, 10]. On the basis of the data presented, it can now be suggested that improved therapy should be achieved when using Irinophore C™ and that the use of Irinophore C™ at efficacious doses will be associated with little or no GI toxicity; thus providing a significant advantage over the currently used clinical formulation.
